# Effects of *Biebersteinia multifida* hydro-ethanol extract on proliferation and apoptosis of human prostate cancer and human embryonic kidney cells

**Published:** 2016

**Authors:** Alireza Golshan, Samira Hassanzadeh, Maryam Mojdekanloo, Zahra Tayarani-Najaran

**Affiliations:** 1 *Medicinal Plants & Natural Products Research Center (MPNPRC), North Khorasan University of Medical Sciences, Bojnurd, Iran*; 2 *Pharmacological Research Centre of Medicinal Plants, School of Medicine, Mashhad, University of Medical Sciences, Mashhad, Iran*; 3 *North Khorasan University of Medical Sciences, Bojnurd, Iran*

**Keywords:** *Biebersteinia multifidi*, *Cytotoxicity*, *Apoptosis*, *Geraniaceae*

## Abstract

**Objective:**

*Biebersteinia* (Geraniaceae) has a history of use in traditional medicine in some countries including Iran. In the present study, cytotoxic and apoptogenic properties of hydro-ethanol extract of *B. multifidi* was investigated on human prostate cancer cell lines (PC3 and DU 145) and human embryonic kidney 293 (HEK293) cells.

**Materials and Methods:**

Cells were cultured in RPMI-1640 medium supplemented with 10% FBS at 37ºC in a humidified atmosphere of 95% air and 5% CO_2_. The root of the plant was macerated with EtOH 70%. Cytotoxic activity of ethanol extract of *B. multifida* was assessed using alamarBlue^®^ assay after 48 hr of treatment. Apoptotic cells were stained with propidium iodide (PI) and detected by flow cytometry (sub-G1 peak).

**Results:**

*B.* *multifidi* had cytotoxic effect on malignant cells and normal HEK293 cells in a dose-dependent manner and significantly decreased the cell viability (IC_50_ values were between 199.2 and 302.9 µg/ml). *B. multifida *increased the sub-G1 peak in flow cytometry histogram of treated PC3 cells compared to control showing the induction of apoptosis and DNA fragmentation.

**Conclusion:**

Due to cytotoxic and apoptotic activity of *B. multifida*, the plant is suggested for further phytochemical analysis and mechanistic evaluation.

## Introduction

Medicinal plants have played a major role in cancer treatment and prevention. Many of natural-based therapeutics have clinical applications based on preliminary screening (Mousavi et al., 2015 ▶). Apoptosis is proposed as the major death pathway by chemotherapeutic agents and there are many natural chemotherapeutic agents which interfere with both extrinsic and intrinsic pathways of apoptosis (Safarzadeh et al., 2014 ▶).

Geraniaceae family has five genera in Iran and *Biebersteinia* is one of them. *Biebersteinia *has four species in the world and *B. multifida *DC. is the only species which is grown in Iran (Janighorban, 2009 ▶). The plant is found in many provinces of Iran and also is found in Caucasia, central Asia, Afghanistan, Iraq and Lebanon. *B. multifida *has yellow flowers and thick root and is of 20-70 cm height (Muellner, 2011 ▶). 

In traditional medicine, the roots of *B. multifida *is used to relieve muscle pain which may be attributed to anti-inflammatory and analgesic properties of the plant (Farsam et al., 2000 ▶). Also, it is used for the treatment of children nocturia, phobia and anxiety (Monsef-Esfahani et al., 2013 ▶). It has been shown that *B. multifida *is effective in rehabilitating bone fractures and decreasing the severity of catatonia following treatment with anti-psychotic drugs (Khakpour and Hadipour, 2008 ▶). Previously published data shows the presence of polysaccharides, peptide, alkaloids like vasicinone, and flavonoids including 7-glucosides of apigenin, luteolin, and tricetin, as well as 7-rutinoside of apigenin and luteolin in this plant (Greenham et al., 2001 ▶; Omurkamzinova et al., 1991 ▶). Vasicinone has been shown to be responsible for some of the pharmacological effects of the plant such as antioxidant and antihemolytic activities (Monsef-Esfahani et al., 2013 ▶; Greenham et al., 2001 ▶). 

There are no reports on the apoptotic activity of *B. multifida *and the aim of this study was to evaluate the cytotoxic and apoptotic activity of the plant on DU 145 and PC3 as two human prostate cancer cell lines.

## Materials and Methods

**Reagents and chemicals**

AlamarBlue^®^ (resazurin) was obtained from Sigma (Saint Louis, MO, USA). RPMI-1640 and fetal bovine serum (FBS) were bought from Gibco (Grand Island, NY, USA). Fluorescent probe propidium iodide (PI) was purchased from Sigma (Steinheim, Germany). All the solvents used for extraction were purchased from Caledon and Scharlau (Spain).

**Plant Materials**

The roots of *B. multifida *were collected from Asadli valley (1340 m height) in Asadli, a village 30 km far from Bojnurd, Nourth Khorasan province, northeast of Iran. The plant was identified by Mr. Imani (Research Center of Natural Products Safety and Medicinal Plants). Voucher specimen (No. npmp45) was deposited in herbarium of North Khorasan University of Medical Sciences. 

The root of the plant (100 g) was dried under standard condition away from light, powdered by electrical grinder and kept in freezer. For preparing the extract of the root, the maceration method with ethanol 70% was used. The obtained filtrate was then dried at 30-40ºC. This yielded 10.2 g of dry extract (10.2%) which was dissolved in dimethylsulfoxide (DMSO) and then was subjected to cytotoxic and apoptosis assays. The concentration of DMSO in sample test was lower than 0.05% (Ahmadzadeh et al., 2014 ▶). 

**Cell cultures**

The human prostate cancer cells (DU 145 and PC3) and human embryonic kidney 293 (HEK293) cells were maintained in RPMI-1640 medium supplemented with 10% FBS, penicillin 100 U/ml, and streptomycin 100 µg/ml at 37ºC in a humidified atmosphere of 95% air and 5% CO_2_. The stock solutions were prepared at 100 mg/ml in dimethylsulfoxide and kept at -20ºC.

For alamarBlue^®^ assay, cells were seeded at 10^4^ cells per well into 96-well culture plates. For assay of apoptosis, cells were seeded at 10^4^ cell per well onto a 24-well plate. For each concentration and time course study, there was a control sample that remained untreated and received the equal volume of DMSO.

**Cell viability**

Resazurin, the active ingredient of alamarBlue^®^ is an indicator which is reduced and converted to resorufin in live and healthy cells. This conversion changes the color of resazurin, a cell permeable redox indicator, from blue to the red and highly fluorescent compound named resorufin (O'Brien et al., 2000 ▶; Boozari et al., 2015 ▶). 

Cells were treated with various concentrations of hydro-ethanol extract of *B. multifida*. After 44 hr incubation, alamarBlue^® ^was added to each well and the absorbance was measured at 570 nm and 600 nm by a Synergy H4 Hybrid Multi-Mode Microplate Reader (BioTek, Winooski, USA Winooski is a city in ChittendenWinooski is a city in Chittenden). 

The IC_50_ values of *B. multifida *extracts were calculated using Graph Pad Software (Graph Pad prism 5 software). Cell viability of three independent experiments each in triplicate was presented as mean ± SD.

**PI Staining**

PI stained treated cells were evaluated by flow cytometry to analyze the apoptotic cells as represented by increased sub-G1 peak in the related histogram and compared to control (Nicoletti et al., 1991 ▶; Motaez et al., 2015 ▶). When stained with quantitative DNA-binding dye PI, DNA small fragments are lost in apoptotic cells incubated in a hypotonic phosphate-citrate buffer containing triton-X100 and will appear to the left of the G1 peak. Briefly, PC3 cells were treated with different concentrations (25, 50, 100 and 200 µg/ml) of hydro-ethanol extract of *B. multifida *for 48 hr. Floating and adherent cells were then harvested and incubated at 4°C for 4 hr in the dark with 400 μl of a hypotonic buffer (PI 50 μg/ml in 0.1% sodium citrate plus 0.1% Triton X-100) before flow cytometric analysis using a FACScan flow cytometer (Becton Dickinson). Here, 10^4^ events were acquired with FACS. 

**Statistical analysis**

One way analysis of variance (ANOVA) and Bonferroni’s *post-hoc* were used for data analysis. All results were expressed as mean ± SD and p values below 0.05 were considered statistically significant.

## Results

**Inhibition of cell viability**

**Figure 1:**
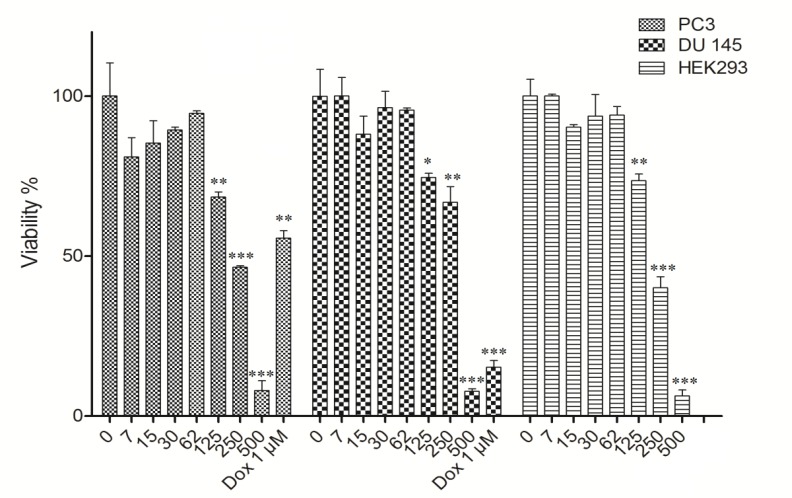
A) Dose-dependent growth inhibition of PC3, DU 145 and HEK-293 cells by hydro-ethanol extract of *B. multifida* (0, 7, 15, 30, 60, 125, 250 and 500 µg/ml) after 48 hr. Viability was quantitated by alamarBlue® assay. The doses inducing IC50 against PC3, DU145 and HEK-293 cells by hydro-ethanol extract of *B. multifida* were 299.5, 302.9 and 199.2 µg/ml, respectively. Doxorubicin (1 µM) was used as the positive control. Results are mean±SD (n = 9). ∗p <0.05, ∗∗p <0.01 and ∗∗∗p <0.001 compared to control

Inhibition of cell viability caused by hydro-ethanol extract of *B. multifida *was examined using alamarBlue^®^ assay. The results showed that the hydro-ethanol extract of *B. multifida *decreased cell viability in a concentration-dependent manner (Figure 1) and significantly decreased the cell viability at the concentration of 125 µg/ml and higher (p< 0.05). The concentrations hydro-ethanol extract of *B. multifida *inducing 50% cell growth inhibition (IC_50_) in PC3, DU 145 and HEK293 cells for are presented in Table 1. Doxorubicin at the concentration of 1 μM was used as the positive control which decreased the cell viability of DU 145 and PC3 cells to 17±3.1% and 55±6.3%, respectively.

**Table 1:** IC_50_ values (µg/ml) for ethanol extract of *B. multifida* in PC3, DU 145 and HEK293 cell lines.

***Cell Line***	***IC***_50 _ ***(*** ***µg/ml)***	***IC***_50 _ ***range***
***PC3***	*299.5 *	*250.3-358.4*
***DU 145 ***	*302.9 *	*250.4-366.5*
***HEK293***	*199.2*	*176.5-224.9*

**Apoptosis induction by hydro-ethanol extract of** ***B. multifida*** **in PC3 and DU 145 cells**

**Figure 2:**
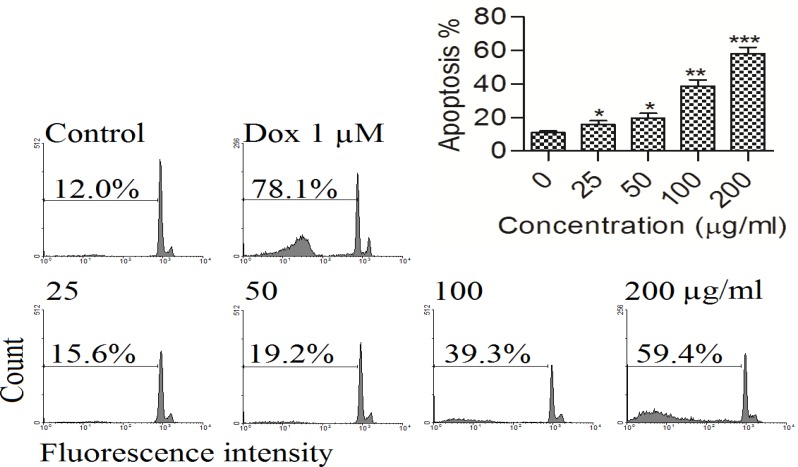
A) Flow cytometry histograms of apoptosis assays by PI method in PC3 cells. Cells were treated with different concentration of hydro-ethanol extract of *B. multifida* (0, 25, 50, 100 and 200 µg/ml) for 48 hr. Sub-G1 peak as an indicative of apoptotic cells, was induced in hydro-ethanol extract of *B. multifida*-treated but not in control cells. Treated cells exhibited a sub-G1 peak in PC3 cells in a concentration-dependent manner that indicates the involvement of an apoptotic process in hydro-ethanol extract of *B. multifida* -induced cell death. Results are representative of three independent experiments. ∗p <0.05, ∗∗p <0.01 and ∗∗∗p <0.001 compared to control

The PC3 cells treated with 25, 50, 100 and 200 µg/ml hydro-ethanol extract of *B. multifida *for 48 hr, induced a sub-G1 peak in flow cytometry histogram compared to untreated control cells (Figure 2) which shows the significant apoptosis induction by the extract (p<0.05 compared to control).

## Discussion

Plants have been served as valuable sources of phytochemicals with various biological effects. Plants and phytochemicals are widely used in treatment of diseases including cancer. Screening of plant and phytochemicals has led to identification of some of the famous groups of chemotherapeutics like taxanes, camptothecins, podophyllotoxins and vinca alkaloids (Fulda, 2010 ▶). In this study, we investigated the putative cytotoxic activity of hydro-ethanol extract of *B. multifida *on PC3, DU 145 and HEK293 cells. After searching for cytotoxic activity of the plant, the apoptotic activity was also explored on PC3 cells. At a range of 0-500 μg/ml, after 48 hr of treatment, the extract decreased the viability of cells in a dose-dependent manner in treated cells. The apoptotic activity was verified in PC3 cells at concentrations of 25, 50, 100 and 200 μg/ml. The subG1 peak in treated cells was increased in a dose-dependent manner which shows the involvement of apoptosis in cytotoxic activity of *B. multifida. *

Literature search shows that *B. multifida* has been investigated for different biological activities. Hashem Dabaghian et al. reported that the plant has anticancer potential as the ethanolic extract of *B. multifida* has the ability to prevent the reverted mutations in antimutagenicity test. They also showed the cytotoxic activity of *B. multifida* in human leukemia pre B-cells (Hashem Dabaghian et al., 2014 ▶). In similar evaluation of methanol extract of 15 Iranian medicinal plants including *B. multifida*, the plant showed cytotoxic activity on MCF-7, HepG2 and WEHI cells (Sahranavard et al., 2009 ▶). In this study, apoptosis-inducing activity of the plant has been showed for the first time.

When evaluated for antioxidant activity, the radical scavenging activities of the essential oil and methanol extract of fruits of *B. multifida *were superior to some other essential oils and extracts tested for this purpose. Additionally, *B. multifida *could effectively inhibit the oxidation of the linoleic acid comparable to butylated hydroxytoluene, curcumine and ascorbic acid (Amiri, 2009). It is reported that different levels of antioxidant and antihemolytic activities of *B. multifida* may be attributed, at least in part, to the presence of phenols and flavonoids in the extracts (Nabavi et al., 2010). Phytochemical studies showed that B. multifida consists of neutral polysaccharides named glucans A, B, and C (Arifkhodzhaev et al., 1985 ▶; Arifkhodzhaev and Rakhimov, 1993 ▶, 1994) and alkaloids (Kurbanov and Zharekeev, 1974 ▶). In this regard, biological activities reported for* B. multifidi *may be associated with the presence of phenols, flavonoids and alkaloid contents of the plant. This study reported the cytotoxic and apoptotic activity of *B. multifida*. The range of IC_50_ values for the plant was between 199.2 and 302.9 µg/ml hydro-ethanol extract. Although there are some reviews which suggested that active cytotoxic plants should have IC50s lower than 100 µg/ml on cancer cells (Taylor et al., 2014 ▶) but there are some active phytochemicals like artemisinin, artesunate and artenimol-R which have been purified from the plants with IC_50_s higher than the acceptable range (Efferth et al., 2011 ▶; Ericsson et al., 2014 ▶; Jansen et al., 2011 ▶). In a cytotoxic evaluation of *Artemisia annua* L., authors reported “The range of IC_50_ values for HeLa cancer cells was 54.1-275.5 μg/ml for dichloromethane extracts and 276.3-1540.8 μg/ml for methanol extracts” (Efferth et al., 2011 ▶). 

Fifty two and fifty three compounds were identified in two independent studies from the essential oil of *B. multifida* after hydrodistillation and gas chromatography which mainly includes (E)-nerolidol, hexadecanoic acid, phytol, and 6,10,14-trimethyl-2-pentadecanone (Ahmadzadeh Sani et al., 2015 ▶; Kamali et al., 2014 ▶; Javidnia et al., 2010 ▶). α-Pinene and 6,1,14-trimethyl-2-pentadecanone were also reported as major components of the essential oil in another study (Akhlaghi et al., 2009 ▶). 

Deregulation of apoptosis in cancer cells has been proposed as the underling mechanism of tumor growth and invasion. Agents interfere with enzymes and proteins which contribute to apoptosis cascade are widely investigated as chemotherapeutics. Apoptosis-enhancing plants and chemicals are classified as putative cancer preventive or therapeutics (Tayarani-Najaran et al., 2014 ▶). Once the cytotoxic activity of *B. multifidi *verified in AlamarBlue^®^ assay, the plant was investigated for potential apoptotic activity. Although the cytotoxic activity of the plant was higher in HEK293 cells, but this is preliminary study and further studies are recommended to compare its malignant and normal cell toxicity. PI staining of the cells treated with *B. multifidi *showed the apoptotic activity of the plant. *B. multifidi *increased the sub G1 peak of treated cells and can be introduced as an apoptosis-enhancing plant. However, further mechanistic investigation will reveal the exact underling mechanisms. Analytical studies to investigate the phytochemicals responsible for biological activity of the plant should also be done in future. In conclusion, although the plant did not show selective cytotoxic activity on cancer cells but it merits further investigation for elucidation of its bioactive cytotoxic compounds. 
